# Intergenerational Classroom Experiences: The Importance of Older Learners’, Students’, and Faculty’s Insights

**DOI:** 10.1093/geroni/igaf122.139

**Published:** 2025-12-31

**Authors:** Chaya Koren, Anna Zisberg, Orit Hirsch-Matsioulas, Sigal Naim

**Affiliations:** University of Haifa, Haifa, HaZafon, Israel; University of Haifa, Haifa, HaZafon, Israel; University of Haifa, Haifa, HaZafon, Israel; The Center for Research and Study of Aging, University of Haifa, Haifa, HaZafon, Israel

## Abstract

Age-inclusive educational practices need research that involves older learners’ perspectives to provide insights about issues impacting intergenerational classroom effectiveness. Accordingly, this study aimed to understand learning/teaching experiences in university intergenerational classroom courses considering older learners’ (aged 65+), along with students’ and faculty’s perspectives. The sample included 11 courses open to older learners. Some with 80 to 150 participants, others with up to 20 participants. Some included more older learners than students. Participants included older learners (n = 11), students (n = 11), and faculty (n = 7). Semi-structured qualitative interviews (n = 29) were audio recorded and transcribed verbatim for data analysis using thematic analysis. Findings highlight the importance of including older learners’ perspectives along with students’ and faculty’s indicating key differences in learning motivations. Older learners sought enrichment, enjoyment, and meaningful engagement without academic obligations, whereas students focused on degree completion and professional development. These contrasting motivations shaped classroom dynamics, influencing perceptions of older learners as either contributing to discussions or disrupting structured learning. Social interactions also played a meaningful role. Older learners form peer connections while perceiving students as part of the campus environment rather than social partners. While both age groups value social engagement, generational differences led to limited cross-generational exchanges mutually shared by both generations. Coinciding with Bronfenbrenner’s ecosystems perspective, findings highlight the importance of including the perspectives of all three stakeholder types for receiving a broader overall perspective on intergenerational classroom experiences. Recommendations include coordinating expectations, providing a teachers’ toolbox, receiving older learners’ feedback, and establishing a suitable older learners-student ratio per course.

